# Supplementary material for: Using iCn3D and the World Wide Web for structure-based collaborative research: Analyzing molecular interactions at the root of COVID-19
Source: bioRxiv. 2020 Jul 6:2020.07.01.182964. Originally published 2020 Jul 2. Preprint. [Version 2] doi: 10.1101/2020.07.01.182964 (PMC7337391; doi:10.1101/2020.07.01.182964)
Supplement: 1 [file NIHPP2020.07.01.182964-supplement-1.pdf]

bioRxiv preprint doi: https://doi.org/10.1101/2020.07.01.182964. this version posted July 6, 2020. The copyright holder for this preprint (which was not certified by peer review) is the author/funder. This article is a US Government work. It is not subject to copyright under 17 USC 105 and is also made available for use under a CC0 license.

SUPPLEMENTS

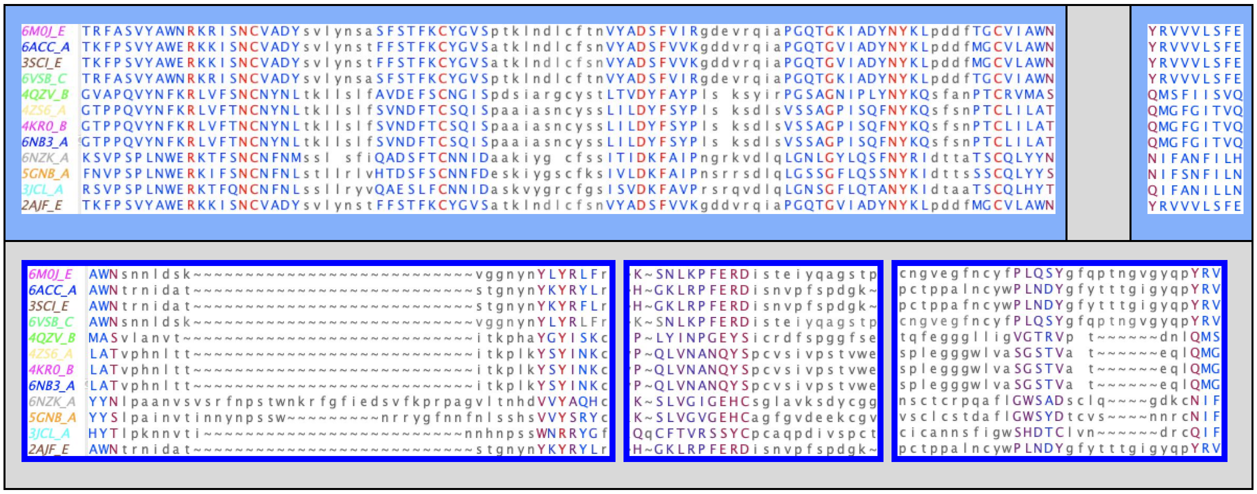

Figure S1 - Structural alignment of known Beta Coronaviruses RBDs: Similar alignment to Figure 3A, with in addition **OC43, HKU1, and MHV**. For clarity we omit additional insertions in the RBM plugin vs. **SARS and MERS** coronaviruses. These insertions increase the RBM size and bring a significant structural complexity due to disulfide bridges. What is remarkable however, is the structural conservation of the RBM SSS despite significant insertions. The first Tyrosine Y133/451 in strand 1 is however, not as conserved as Y135/453, as in, for example, HKU1 or OC43 coronaviruses (PDBids 5GNB and 6NZK) where it is replaced by a Valine. This broader coronaviruses sequence alignment is visualized in iCn3D (Link 3). In the structure of a mouse hepatitis virus (MHV) (PDBid: 3JCL), the 3D superposition also shows a structurally conserved RBM strand 1 where the sequence **WNR** matches the **Y/VxY** pattern location, yet an evolutionary-based sequence alignment of beta coronaviruses (CD21470) finds a **VVY** match as in HKU1 and OC43, located in sequence right after the (PSS**WNR**RYGF) sequence conserved in OC43 and HKU1. The disagreement is most likely the result of the region being poorly defined in the electron density map, as stated by the authors of the structure and seen in the following figure (Figure S2) Whether an artefact of the EM structure or a sequence shift during evolution, the RBM structural pattern (antiparallel strand1-strand2-flanking loop) is conserved across all know beta coronaviruses.

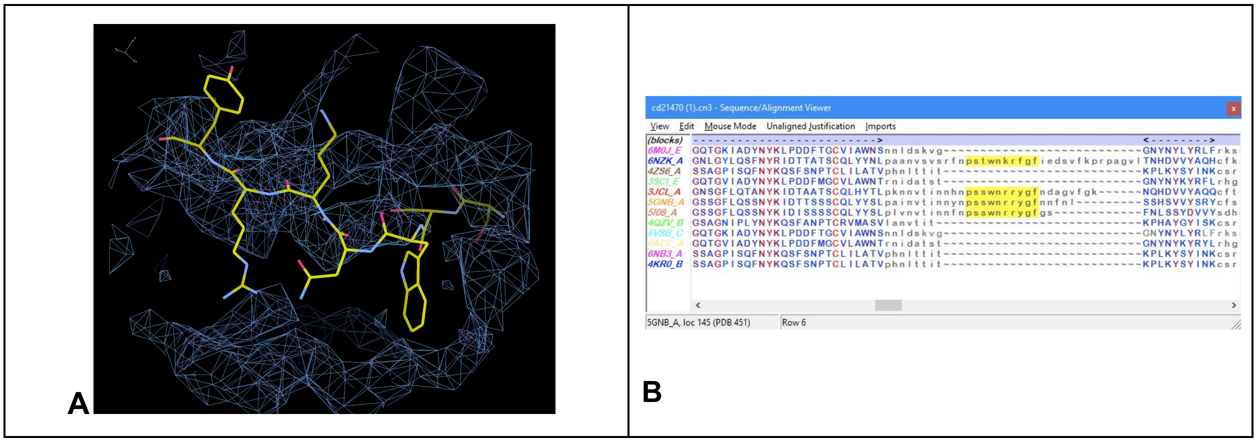

bioRxiv preprint doi: https://doi.org/10.1101/2020.07.01.182964. this version posted July 6, 2020. The copyright holder for this preprint (which was not certified by peer review) is the author/funder. This article is a US Government work. It is not subject to copyright under 17 USC 105 and is also made available for use under a CC0 license.

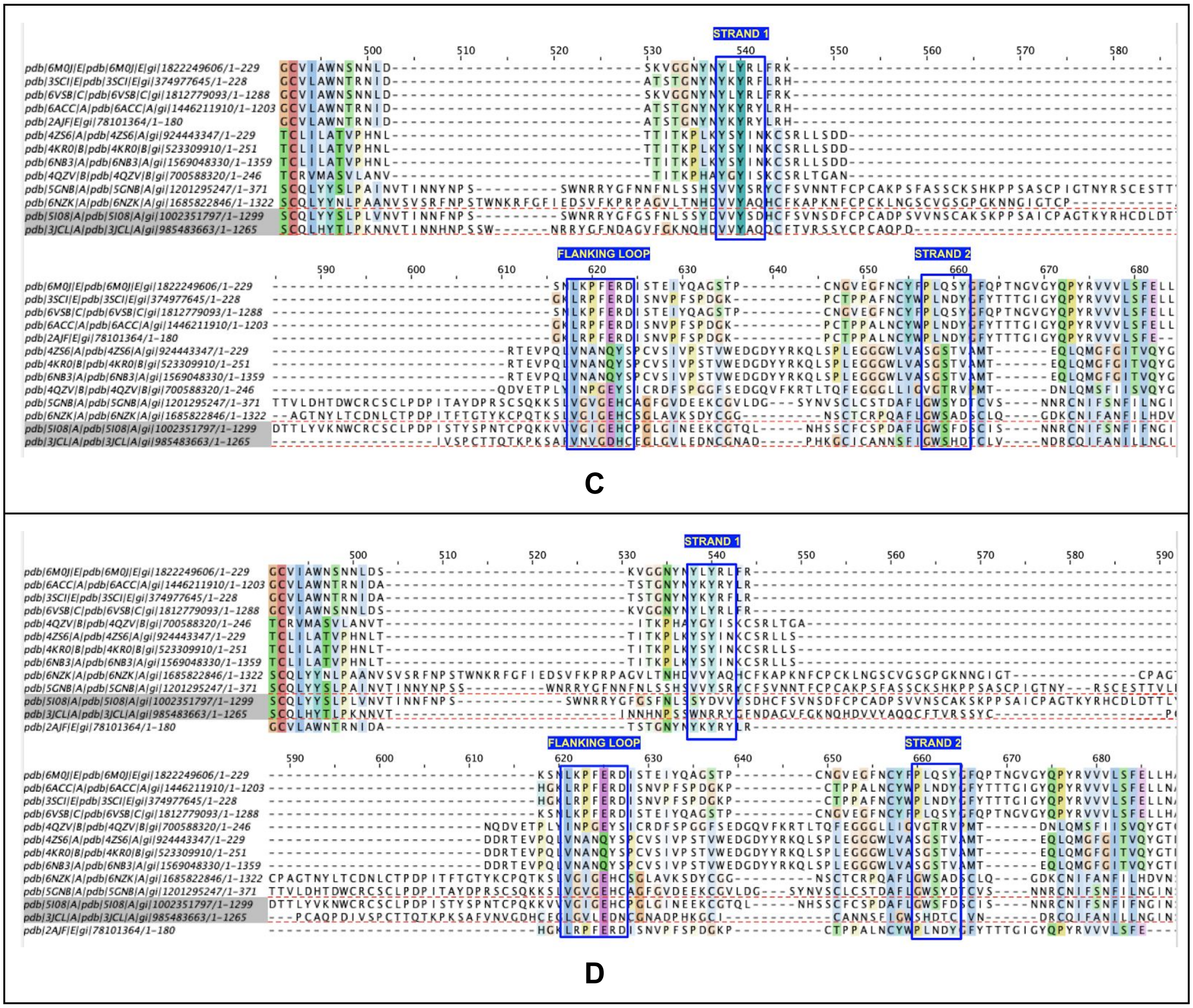

Figure S2 - RBM subdomains of beta coronaviruses

A) electron density of MHV (Murine Leukemia Virus) structure 3JCL in the RBM region. The sequence **WNR** was fitted in place of VV**Y**

B) Beta Coronaviruses CDD (CD21470**)** showing the conservation of the PSSWNRRYGF sequence in OC43 and HKU1 as well as MHV

C)-Optimized Sequence alignment of beta coronaviruses RBMs taking into account homologous patterns observed in the CDD (B) where Strand 1 aligns Yx**Y** motif of SARS/MERS vs. VV**Y** for OC43/HKU1/HKU4/MHV. In Strand 2 similarly a GWS pattern matches in these four viruses the PLQ/N sequence in SARS. Similarly the GD-box like flanking loop, between strand 1 and strand 2 aligns GxxE between OC43/HKU1/HKU4/MHV

D)-Optimized structure alignment of beta coronaviruses RBMs where in 3JCL WNR was fitted (see A) and aligns with other structures VVY in strand 1', in strand 2 the structure presents a shift of 2 residues of GWS when compared to other structures C) . The HKU1 (PDB 5I08) structure, in the same way shifts by 3 residues strand 1 matching VV**Y** with the preceding SYD residues, yet strand 2 and the flanking loop are well positioned.

bioRxiv preprint doi: https://doi.org/10.1101/2020.07.01.182964. this version posted July 6, 2020. The copyright holder for this preprint (which was not certified by peer review) is the author/funder. This article is a US Government work. It is not subject to copyright under 17 USC 105 and is also made available for use under a CC0 license.

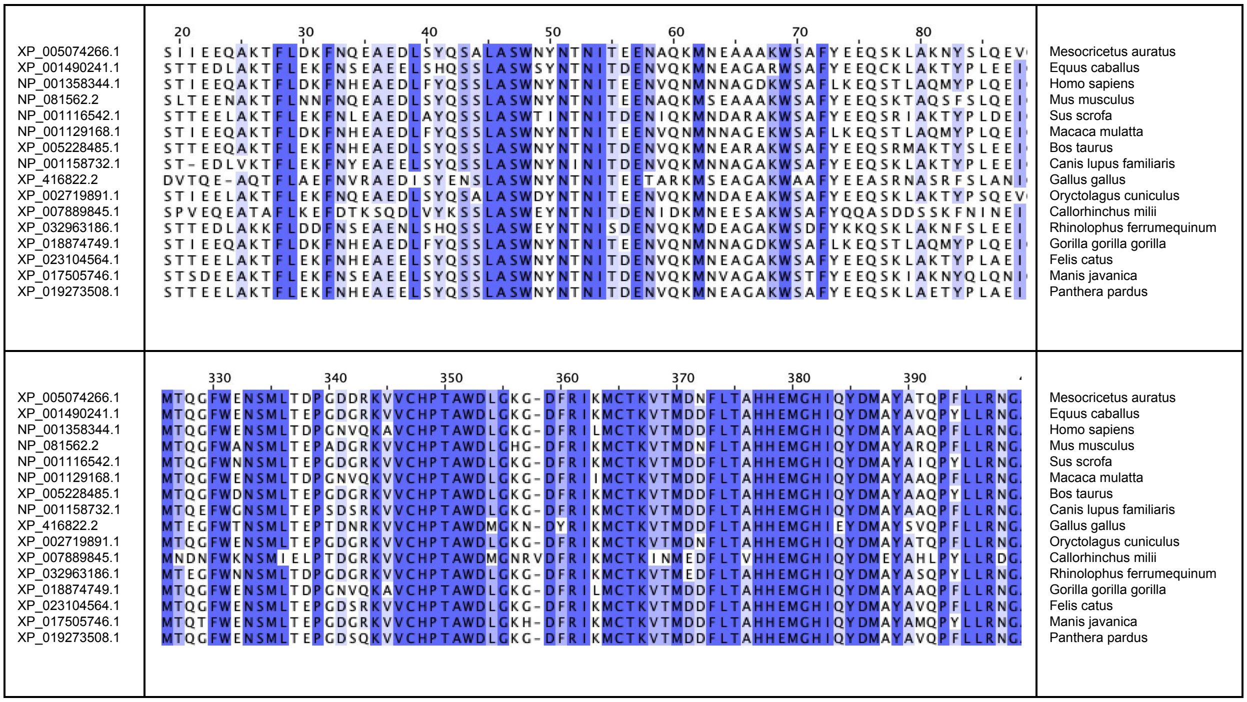

Figure S3 - ACE2 sequences of selected mammals. Two subdomains contain the residues forming the receptor binding site (RBS) of ACE2, 1. N terminal domain (res 19-90) and 2. subdomain (res. 326-400) [see Figure 2-3-4] for more details.

Free energy calculations

ΔΔG values were computed using the Free Energy Perturbation slow growth calculation approach as described in [1] using Gromacs 5.1.4 [2] and the PMX package [3] considering the "unbound" geometries (instead of the "unfolded" states as described in [1]) The ΔΔG values were computed in the forward and backwards direction. The values reported are the average of the two. The Amber99sb force field was used for all FEP calculations. Initial coordinates were obtained from the RCSB and preprocessed using Yasara version 20 [4] and the "Clean All" macro using the NOVA2 force field, followed by an energy minimization using the same force field and default parameters using the Options / Energy Minimization macro. The coordinates were then subjected to the regular relaxation in a solvent bath as described in [1] before the molecular dynamics trajectories were collected.

[1] https://www3.mpibpc.mpg.de/groups/de_groot/cecam2015/peptide_mutation/
[2] http://manual.gromacs.org/documentation/5.1.4/
[3] https://github.com/dseeliger/pmx/
[4] http://www.yasara.org/
